# A scoping review of imatinib-induced testicular toxicity and male fertility impairment

**DOI:** 10.3389/fendo.2026.1811653

**Published:** 2026-05-14

**Authors:** Xia Ji, Mohd Faizal Ahmad, Mohd Helmy Mokhtar, XiaoYing He, Abdul Kadir Abdul Karim

**Affiliations:** 1 Department of Gynaecology and Obstetrics, Inner Mongolia Baogang Hospital, Baotou, Inner Mongolia, China; 2 Department of Obstetrics & Gynecology, Faculty of Medicine, Universiti Kebangsaan Malaysia, Kuala Lumpur, Malaysia; 3 Department of Physiology, Faculty of Medicine, Universiti Kebangsaan Malaysia, Kuala Lumpur, Malaysia; 4 School of Life Science and Technology, Inner Mongolia University of Science and Technology, Baotou, China

**Keywords:** fertility preservation, imatinib mesylate, spermatogenesis, testicular diseases, testis/drug effects

## Abstract

**Background:**

Imatinib, the first-generation tyrosine kinase inhibitor (TKI), has been widely adopted as frontline therapy for chronic myeloid leukemia (CML) and gastrointestinal stromal tumors (GIST). Growing evidence indicates potential gonadotoxic effects, raising concerns about its long-term impact on male fertility.

**Objective:**

This scoping review was undertaken to synthesize preclinical and clinical evidence on imatinib-induced reproductive toxicity in males, with emphasis on mechanisms, dose- and age-dependent susceptibility, and reversibility of testicular injury.

**Methods:**

A comprehensive literature search was conducted in PubMed, Scopus, and Web of Science following PRISMA-ScR guidelines. Twenty studies published between 2003 and 2025 were included.

**Results:**

Across animal and human studies, inhibition of Proto-oncogene c-KIT (c-KIT) and Platelet-Derived Growth Factor Receptor (PDGFR) signaling was consistently observed, leading to germ cell apoptosis, impaired spermatogonial proliferation, and disruption of the blood–testis barrier (BTB). Dose-dependent reductions in testosterone and sperm density were documented, with partial recovery after drug discontinuation in several models. However, neonatal exposure was more often associated with persistent or irreversible testicular damage.

**Conclusion:**

Imatinib exerts gonadotoxic effects through inhibition of c-KIT/PDGFR signaling, disruption of the BTB, and dysregulation of the hypothalamic–pituitary–gonadal axis in a dose- and age-dependent manner. Although partial recovery is possible after withdrawal, neonatal and prepubertal exposures carry a high risk of irreversible impairment. These findings highlight the need for systematic fertility counseling and preservation in adolescent and reproductive-age males, in line with the 2025 European LeukemiaNet (ELN) recommendations.

## 1 Introduction

Leukemia is a hematologic malignancy characterized by the abnormal proliferation of hematopoietic stem cells (HSCs), resulting in impaired hematopoiesis and systemic dysfunction. It accounted for 3.2% of cancer-related mortality worldwide in 2022 (1, 2). Chronic myeloid leukemia (CML), defined by the Philadelphia chromosome translocation t (9;22) that generates the BCR-ABL1 fusion oncogene, shows a male predominance (male-to-female ratio: 1.16:1) (3–6). Although CML predominantly affects adults, pediatric and adolescent cases are uncommon, and because these patients receive long-term treatment during critical periods of growth and development, they present unique clinical challenges (7, 8). Since the Food and Drug Administration (FDA) approval of imatinib in 2001, this first-line tyrosine kinase inhibitor (TKI) has transformed the prognosis of CML, with 10-year survival rates exceeding 80% (9). Nevertheless, concerns regarding its potential gonadotoxicity have increasingly been recognized.

Evidence from both experimental and clinical studies has suggested an adverse impact of imatinib on testicular function, manifested as reduced sperm count, compromised sperm quality, and, in some cases, transient infertility (10, 11). These effects have been attributed to the inhibition of cell division in germ cells, which are highly sensitive to both cytotoxic and targeted agents. Clinical observations further indicate that male CML patients treated with imatinib for 24 months exhibited up to a 70% reduction in sperm density (12, 13).

Despite the clinical relevance, several knowledge gaps remain. First, the inhibition of key regulators of spermatogenesis, particularly Proto-oncogene c-KIT (c-KIT) and Platelet-Derived Growth Factor Receptor (PDGFR) receptors, has been implicated, yet the precise consequences for the seminiferous epithelium remain insufficiently defined (14, 15). Second, the rising incidence of CML in adolescents and young adults underscores the need to clarify age specific reproductive risks, particularly given the prolonged treatment duration and the developmental sensitivity of the reproductive system (15), Third, although progress has been made in delineating the mechanisms of testicular toxicity, clinically validated biomarkers capable of predicting individual susceptibility are lacking, limiting the ability to identify high risk patients at an early stage and delaying the timely implementation of fertility preserving interventions.

This scoping review therefore synthesizes available evidence along three principal dimensions: (1) molecular mechanisms underlying imatinib-induced disruption of spermatogenesis; (2) dose- and age-dependent effects on testicular structure and function; and (3) clinical challenges associated with fertility preservation in pubertal and reproductive age males. By integrating these domains, the review seeks to provide a framework for future therapeutic strategies that safeguard reproductive health while maintaining oncologic efficacy.

## 2 Materials and methods

**Figure 1:**
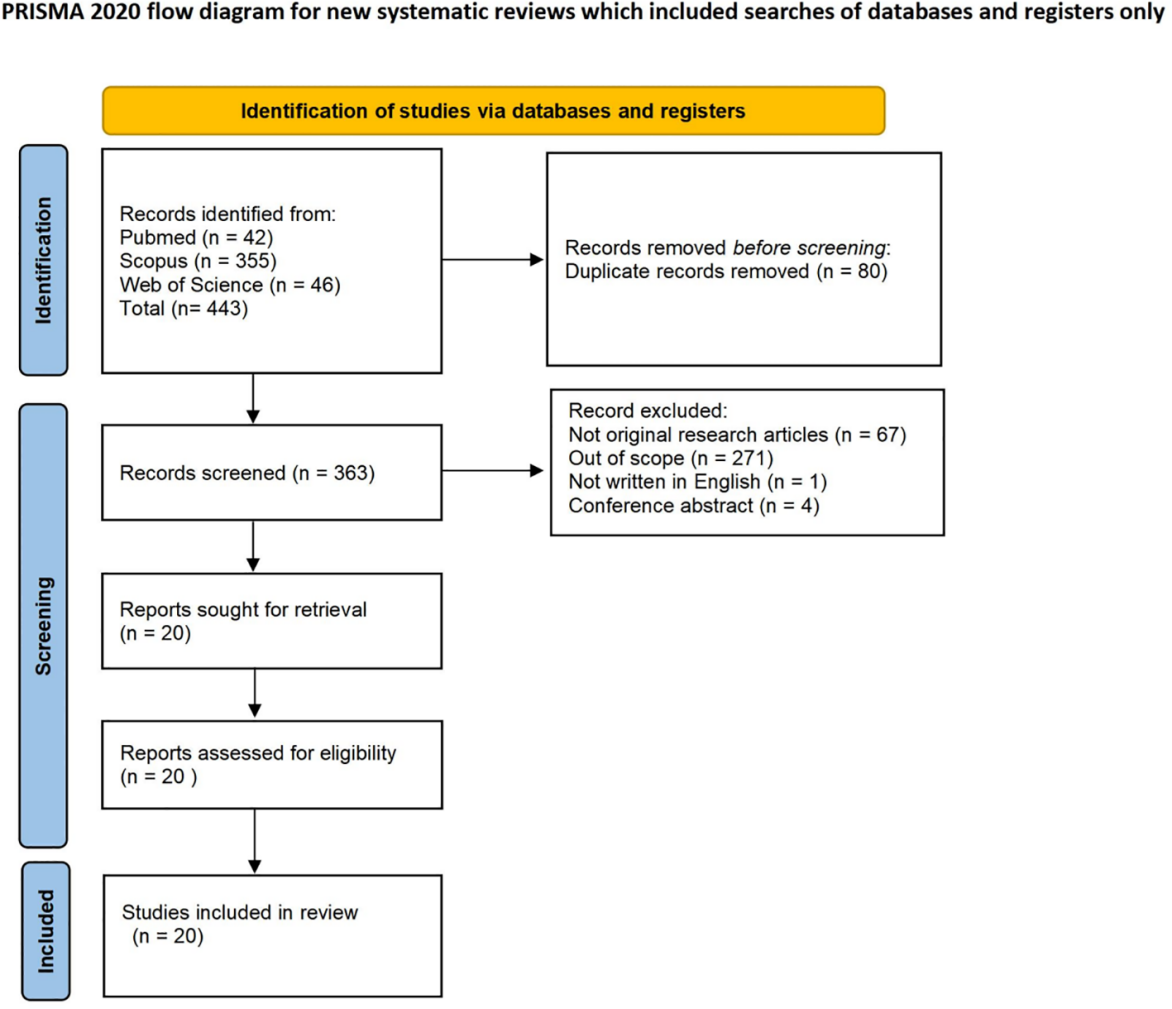
Flow diagram of the study selection process.

This scoping review was conducted in accordance with the PRISMA-ScR guideline for scoping reviews. The flow diagram of the study selection process is shown in **Figure 1**. On October 1, 2025, a comprehensive search was undertaken in three electronic databases; PubMed, Scopus, and Web of Science to identify studies investigating the impact of imatinib on male reproductive function.

The search strategy included the following terms: (“Imatinib” OR “Imatinib mesylate”) AND (“Testis” OR “Testes” OR “Spermatogenesis” OR “Sperm” OR “Male fertility” OR “Leydig cells” OR “Sertoli cells” OR “Male reproductive toxicity”). Reference lists of selected studies were also scrutinized to identify additional relevant articles.

The search strategy was adapted for each database using appropriate controlled vocabulary and free-text terms. In PubMed, Medical Subject Headings (MeSH) terms were used where applicable, combined with keywords. Equivalent search strategies were applied in Scopus and Web of Science using database-specific field tags. Boolean operators (“AND”, “OR”) were used to combine search terms. No restrictions on study design were applied. The detailed search strategies for each database are provided in **Supplementary Table 1**.

Minor variations in the number of retrieved records may occur due to database updates and differences in indexing across platforms.

In keeping with the standard methodology of scoping reviews, no formal quality appraisal or risk-of-bias assessment was undertaken, as the objective was to provide an overview of the available evidence rather than to evaluate the internal validity of individual studies.

### 2.1 Eligibility criteria and study selection

Studies were included if they fulfilled the following criteria: (1) original research addressing the impact of imatinib on male reproductive health, and (2) the use of *in vitro* models, animal experiments, or human subjects. Exclusion criteria were: (1) non-original articles (including reviews, commentaries, editorials, conference abstracts, and letters without primary data) and (2) non-English articles.

Two reviewers (X. Ji and X.Y. He) independently screened the titles and abstracts of all retrieved records. Full-text articles were subsequently assessed for eligibility based on the predefined inclusion and exclusion criteria. Discrepancies were resolved through discussion, and where consensus was not achieved, a third reviewer (A. K. A. Karim) adjudicated the decision.

### 2.2 Data extraction

All references were managed using EndNote 21 (Clarivate Analytics, Philadelphia, PA, USA). Duplicates were removed using both automated and manual methods. Data extraction was performed independently by two reviewers (Ji and He) using a standardized form. Extracted information included first author, publication year, study model, imatinib dosage, treatment duration, and key reproductive outcomes.

### 2.3 Data synthesis

Given the substantial heterogeneity across included studies, including differences in species, experimental design, dosing regimens, exposure duration, and outcome measures, quantitative synthesis was not appropriate. Therefore, findings were summarized descriptively.

### 2.4 Article selection

A total of 443 records were identified through database searching. After removal of 80 duplicates, 343 records were excluded (67 non-original articles, 271 out of scope, one non-English article, and four conference abstracts). The remaining 20 full-text articles were assessed for eligibility, all of which met the predefined inclusion criteria and were included in the final synthesis (**Figure 1**).

### 2.5 Research characteristics

This review encompassed 20 experimental and clinical studies published between 2003 and 2025, covering evidence from murine, rat, zebrafish, human cohorts, as well as cellular and ex vivo testicular models. To better delineate patterns and mechanistic progression, the results are organized according to a “model progression–mechanistic elaboration” framework. **Tables 1** and **2** summarize the dose-dependent effects of imatinib on spermatogenesis, testicular architecture, and hormonal regulation in mouse and rat models respectively. **Table 3** provides rodent–human age equivalence to contextualize exposure windows. **Table 4** highlights the unique contribution of zebrafish studies in characterizing early developmental toxicity and potential transgenerational phenotypes. **Table 5** compiles evidence from human investigations, revealing age and treatment-window specific vulnerabilities in reproductive function. **Table 6** presents findings from cellular and ex vivo tubule systems, in which direct actions of imatinib on spermatogonial stem cells, Sertoli cells, and Leydig cells were elucidated at molecular and cellular levels. Finally, **Table 7** integrates these multi-tiered findings into a stage-based framework of spermatogenesis, encompassing germ cell migration, Spermatogonial stem cell (SSC) maintenance, meiotic progression, sperm maturation, and interstitial homeostasis. This reconstruction enables the sequential mapping of vulnerable nodes and interconnections across stages, thereby providing a consolidated trajectory of imatinib-induced reproductive toxicity.

**Table 1:** Summary of studies evaluating imatinib - induced reproductive toxicity in mice models.

Author	Murine species/age of treatment/dosage	Key Ffindings	Conclusion
Chang et al., 2021 (19)	Kunming mice, 8 weeks old males Dose: 60, 90, 120 mg/kg/day × 8 weeks (i. p.) Control: Saline	• Impaired sperm quality (↓ count, ↑ deformity) • BTB disruption • ↑ Germ cell apoptosis • Hormonal imbalance (LH↑, E_2_↑)	Imatinib reduces male fertility by Penetration and disruption the blood-testis barrier, induction of apoptosis in meiotic germ cells, and disrupting seminiferous epithelium.
Prasad et al., 2010^a^ (18)	Swiss albino, 9–12 weeks old males Dose: 50, 75, 100 mg/kg×5 days (i. p.) Control: Distilled water	• Sperm count ↓ • Sperm motility ↓ • Abnormal sperm morphology ↑ • Germ cell cytotoxicity	Imatinib induces dose-dependent impairment of sperm parameters, including reduced sperm count, decreased motility, and increased morphological abnormalities, reflecting germ cell toxicity. These effects are largely reversible after drug withdrawal.
Prasad et al., 2010^b^ (12)	Swiss albino, 9–12 weeks old males Dose: 50, 75, 100 mg/kg×5 days (i. p.) Control: Distilled water	• Germ cell sloughing • Seminiferous epithelial height ↓ • Sperm cell number ↓ • Structural disruption of seminiferous tubules	These histopathological alterations are dose-dependent and largely reversible after drug withdrawal, with delayed recovery at higher doses.
Prasad et al., 2011^c^ (17)	Swiss albino, 9–12 weeks old males Dose: 50, 75, 100 mg/kg×5 days (i. p.) Control: Distilled water	• Intratesticular testosterone ↓ • LDH (marker of testicular cytotoxicity) ↑ • Evidence of Leydig cell dysfunction	Imatinib decreased testosterone and increased LDH during weeks 2–5, with both parameters returning to normal by week 10; effects were dose-dependent but reversible.
Schultheis et al., 2012 (16)	C3H/HeJ, 5-week-old males Dose: 150 mg/kg/day×2 months (water) Control: Saline	• No significant effects on spermatogenesis or testicular structure	Imatinib at therapeutic doses does not affect testicular function.

References (21–23) represent serial publications from the same research group using the same Swiss albino mouse model.

Hormones: FSH, Follicle-Stimulating Hormone; LH, Luteinizing Hormone; PRL, Prolactin; E₂, Estradiol; T, Testosterone; P, Progesterone.

Structural & Administration Terms: BTB, Blood -Testis Barrier; i.p., Intraperitoneal.. Biochemical & Oxidative Stress Markers: CAT, Catalase; GSH, Glutathione; SOD, Superoxide Dismutase; MDA, Malondialdehyd. Physiological Indices: Organ index = organ weight (mg)/body weight (g).

**Table 2:** Summary of studies evaluating imatinib-induced reproductive toxicity in rat models.

Author	Murine species/age of treatment /dosage	Key findings	Conclusion
Suzan et al., 2021 (20)	SD rats; Pregnant females treated on gestation days 1–8; male offspring analyzed at PND 60 Dose: 20, 40, 60 and 80 mg/kg× 8 days (i. p.)	• ↓ sperm count • Mild impairment of testicular structure • Altered chromatin remodeling (protamine/histone imbalance)	Prenatal imatinib exposure in rats caused dose-dependent sperm reduction and mild testicular changes in male offspring, with preserved germ cell maturation.
Al-Allaf et al., 2021 (14)	Albino rats; Neonates (PND1-10); males Dose: 200 mg/kg/day× 10 days (oral gavage)	• Severe testicular injury • ↑ germ cell apoptosis • Impaired spermatogenesis	Early neonatal exposure to imatinib induces severe testicular injury with age-dependent susceptibility, showing peak damage at PND40–70 and only partial recovery by PND140.
Nurmio et al., 2007 (23)	SD rats; Neonates (PND5), males Dose: 50/150 mg/kg×3 days (oral gavage)	• ↑ germ cell apoptosis • Impaired gonocyte migration and SSC formation • ↓ testicular growth	Early postnatal exposure disrupted gonocyte migration and SSC pool formation, reduced testis weight and cord length, induced apoptosis with long-term increases in LH/FSH, while adult testicular morphology and sperm counts were preserved.
Nurmio et al., 2008 (24)	SD rats; Neonates (PND5-7), males Dose: 150 mg/kg/day × 3 days (oral gavage)	• ↓ testis and epididymis weight • ↓ litter size • ↑ LH/FSH	Early neonatal imatinib exposure reduced testis/epididymis weight and litter size with persistent LH/FSH elevation, while adult testicular structure, testosterone, and sperm counts were preserved.
Tauer et al., 2014 (22)	Wistar rats; 4-14week Dose: 1–2 mM × 10 weeks (oral gavage)	• Slight ↓ testosterone (high dose) • Stable inhibin B	Chronic imatinib exposure in juvenile rats caused a slight, non-significant testosterone decrease at high dose, with stable inhibin B, suggesting no evident testicular toxicity.
Yaghmaei et al., 2009 (21)	Wistar rats; Adult males Dose: 6, 9, 12 mg/kg × 30 days (oral gavage)	• ↓ spermatogonia +• ↓ spermatocytes • ↓ Sertoli cells • ↓ Leydig cells • ↓ seminiferous tubule diameter • ↓ sperm count+ ↑ tunica albuginea thickness	Imatinib exerts dose-dependent adverse effects on spermatogenesis, with associated reductions in sperm count, cell populations, and seminiferous tubule structure.

Hormones: T, Testosterone; FSH, Follicle-Stimulating Hormone; LH, Luteinizing Hormone; PDGF, Platelet-Derived Growth Factor; SCF, Stem Cell Factor; Biomarkers & Oxidative Stress Indicators: TBARS, Thiobarbituric Acid Reactive Substances; 8-OHdG, 8-Hydroxy-2′deoxyguanosine; GSH, Glutathione. Enzymes: SOD, Superoxide Dismutase; CAT, Catalase; P450scc, Cytochrome P450 side-chain cleavage enzyme.

Experimental Terms & Timepoints: NR, Not Reported; PND5 -7, 5 -7 days after birth; PND40 -70, 40 -70 days after birth; PND77, 77 days after birth.

**Table 3:** Age equivalence of rats and mice to human developmental stages.

Species	Preterm	Newborn	Infant	Child	Adolescent
Rat	0–4 days	0–10 days	1.5–3 weeks	3–6 weeks	7–11 weeks
Mouse	0–4 days	0–10 days	1.5–3 weeks	3–5 weeks	5–7 weeks
Human	–	0–28 days	1–23 months	2–12 years	12–16 years

**Table 4:** Summary of study evaluating imatinib-induced reproductive toxicity in zebrafish models.

Author	Species/age of treatment /dosage	Key findings	Conclusion
Ahmadi et al., 2019 (26)	Zebrafish,6 months Dose: IM-BSN once, twice and three times daily × 30 days Negative control: pure BSN	• ↓ Sperm quality (density and motility) • ↓ Fertility and fecundity • Suppressed folliculogenesis • Morphometric abnormalities in offspring	Imatinib may have negative effects on the reproductive system.

Experimental Terms & Interventions:.

BSN, Brine Shrimp Nutrition; IM-BSN, Imatinib–Brine Shrimp Nutrition.

**Table 5:** Summary of studies evaluating imatinib-induced reproductive toxicity in males with chronic myeloid leukemia.

Reference	Age/dose/control	Key findings	Conclusion
Gambacorti-Passerini et al., 2003 (27)	38 CML male patients (28–84 y) Dose: 400–800 mg/day × 6–31 months (oral) 21 patients (self-controlled, pre -vs during treatment)	• ↓ testosterone (total & free) • ↑ progesterone and 17-OHP • Gynecomastia (~18%)	Imatinib reduces testosterone and increases progesterone/17-OHP, causing gynaecomastia in ~18% of patients.
Ahmed et al., 2021 (28)	42 CML male patients (23–68 y) Dose: 400 mg/day × >2 years (oral) control: 45 healthy male volunteers	• ↓ testosterone • ↑ LH and FSH (compensatory)	Imatinib lowers testosterone with compensatory LH/FSH rise, while Hb and WBC remain stable.
Chang et al., 2017 (13)	48 CML male patients (15–51 y) Dose: 400 mg/day × 3–96 months, (oral) control: 50 healthy male volunteers Positive control: 10 infertile men	• Impaired sperm parameters (↓ count, motility, survival) • Hydrocele observed in a substantial proportion of patients (~40%)	Imatinib impairs sperm parameters, while reproductive organ structure and hormone levels remain within normal ranges.
Nesr G et al., 2024 (29)	11 CP-CML male patients (23–45 y) Dose: Imatinib, Nilotinib, Dasatinib, Bosutinib, duration 2.5–16.5 years control: pre-treatment paired semen samples	• Preserved sperm concentration and motility • Abnormal morphology in some cases	Long-term TKI therapy did not impair semen concentration or motility, although abnormal morphology was observed in some cases, with preserved fertility potential.

Hormones: FSH, Follicle-Stimulating Hormone; LH, Luteinizing Hormone; T, Testosterone. Biomarkers & Oxidative Stress Indicators: DFI, DNA Fragmentation Index; GSH, Glutathione; MDA, Malondialdehyde. Clinical & Hematological Parameters: WBC, White Blood Cells; Hb, Hemoglobin; CML-CP, Chronic Myeloid Leukemia -Chronic Phase.

**Table 6:** Summary of studies evaluating imatinib-induced effects on male reproductive parameters in cell-based models.

Reference	Cell type/dose/control	Key findings	Conclusion
Kheradmand et al., 2016 (30)	TM3 Leydig cell line Dose: 2. 5, 5, 10, 20 μM×2, 4, or 6 days	• ↓ cell viability • ↑ PDGF levels • SCF unchanged	Imatinib reduces TM3 Leydig cell viability in a dose-dependent but not time-dependent manner, with increased PDGF levels and no significant change in SCF expression.
Hashemnia et al., 2016 (31)	TM4 Sertoli cell line Dose: 2. 5, 5, 10, 20 μM×2, 4, or 6 days	• ↓ Cell viability (dose-dependent) • No significant change in PDGF and SCF levels	Imatinib reduces Sertoli cell viability in a dose-dependent manner, while PDGF and SCF levels remain largely unchanged, suggesting cytotoxic effects independent of growth factor modulation.
Heim et al., 2011 (10)	GS cells (SSC lines) Dose: 1 μM × 9–10 days	• ↓ differentiated spermatogonia • ↓ total cell expansion • SSC self-renewal unaffected	Imatinib selectively reduced differentiated spermatogonia and overall culture expansion, while sparing spermatogonial stem cell self-renewal, proliferation, and differentiation capacity.
Eggert et al., 2024 (32)	Seminiferous tubule segments and male mGSCs isolated from DBA/2J mouse testes Dose: 0, 1, 10, 100 μM × 24–72 hours	• ↓ cell proliferation and DNA synthesis • ↓ c-KIT^+^ germ cell population • No direct apoptosis induction	Imatinib reduced spermatogenic cell proliferation and survival ex vivo and *in vitro* by inhibiting SCF/c-KIT signaling, decreasing DNA synthesis and c-KIT expression, and limiting mGSC expansion, without directly inducing apoptosis.

TM3, Leydig Cell line; TM4, Sertoli Cells; MEF, Mouse Embryonic Fibroblast; GS Cells, Germline Stem Cells; SSC, Spermatogonial Stem Cell; mGSC, Male Germline Stem Cell. Signaling Molecules & Receptors: PDGF, Platelet-Derived Growth Factor; SCF, Stem Cell Factor; KIT, c-KIT Receptor. Biochemical & Stress Markers: ROS, Reactive Oxygen Species; MDA, Malondialdehyde; LDH, Lactate Dehydrogenase; GSH, Glutathione.

Experimental Tools & Markers: GFP, Green Fluorescent Protein; BrdU, 5-Bromo-2'-deoxyuridine; RA, Retinoic Acid.

**Table 7:** Summary of dose-dependent effects of imatinib across spermatogenic stages and testicular compartments.

Stage/Cell Type	Key Function	Dose-dependent Effects	Detection Methods & Indicators
Postnatal germ cell migration stage (gonocyte/SSC localization)	Migration of gonocytes to basement membrane and SSC pool establishment	• Low dose (50 mg/kg, rat): impaired migration; central aggregation; ↓ peripheral SSC localization	• Imatinib disrupts SSC niche establishment by impairing germ cell migration and basement membrane integrity (14, 23)
		• High dose (200 mg/kg, rat): severe central aggregation; germ cell depletion; ↓ seminiferous tubule diameter; basement membrane disruption	
Spermatocyte meiotic stage (SCC)	SSC self-renewal, proliferation, and differentiation	• Low dose (1–10 μM, *in vitro*): ↓ DNA synthesis; ↓ S-phase fraction; ↑ apoptosis; ↓ c-KIT^+^ cells; ↓ colony formation (30–70%)	• SCF/c-KIT pathway inhibition leads to dose-dependent suppression of SSC proliferation and surviva (23)
		• High dose (100 μM): proliferation arrest (no S-phase); widespread apoptosis; ↓ colony formation (>90%); irreversible SSC loss• irreversible stem cell loss	
Spermatocyte meiotic stage (spermatocytes)	Germ cell proliferation and meiotic differentiation	• Low dose (60 mg/kg, mouse): ↓ sperm count and motility; slight ↑ abnormalities; mild BTB disruption; moderate apoptosis	• BTB disruption and activation of apoptotic pathways impair meiotic progression (19)
		• High dose (120 mg/kg): marked ↓ sperm count/motility; ↑↑ abnormalities; complete BTB breakdown; pronounced apoptosis	
Testicular interstitial support stage (Leydig/vascular compartment)	Hormonal regulation and structural support	• Low dose (150 mg/kg, rat): no significant changes in Leydig cell morphology or PDGF/SCF signaling	• Interstitial compartment shows relative resistance, with delayed and partially reversible alterations (23)
		• High dose (200 mg/kg): interstitial edema; ↑ Leydig cell number (PND70); partial recovery by PND140	

Cell Types & Germline Markers: SSC, Spermatogonial Stem Cell; mGSC, Male Germline Stem Cell; c-KIT, Receptor Tyrosine Kinase; Ki67, Cell Proliferation Marker; PCNA, Proliferating Cell Nuclear Antigen; Mfsd2a, Membrane Transport Protein. Histological & Imaging Techniques: H&E, Hematoxylin and Eosin; IHC, Immunohistochemistry; IF, Immunofluorescence; TEM, Transmission Electron Microscopy; WB, Western Blot; TUNEL, Apoptosis Assay. Signaling Molecules & Growth Factors: PDGF, Platelet-Derived Growth Factor; SCF, Stem Cell Factor; RA, Retinoic Acid. Apoptosis & Cell Death Markers: Bax, Bcl-2-associated X Protein; Bcl-2, B-cell Lymphoma 2; Caspase-3, Cysteine-aspartic Protease 3. Molecular Biology Techniques: RT-PCR , Reverse Transcription PCR. Structural & Developmental Terms: BTB, Blood -Testis Barrier; BM, Basement Membrane; 3β-HSD1, 3β-Hydroxysteroid Dehydrogenase 1; PND, Postnata Day.

#### 2.5.1 Reproductive effects of imatinib in mouse models

There were 5 studies obtained across murine models as summarized in **Table 1**. There were one each using Kunming (6) and C3H/HeJ (16), while there were three using Swiss albino strains (12, 17). Dosing regimens of 50–150 mg/kg for 5 days to 2 months were looked at. Intraperitoneal injection was most frequently applied (12, 17–19), whereas one study relied on oral administration via drinking water (16).

Chang et al. (2021) demonstrated that intraperitoneal exposure to 60–120 mg/kg for 8 weeks was associated with reductions in testis index and sperm counts, detachment of the seminiferous epithelium, BTB disruption, and increased Bax and estradiol (19). In Swiss albino mice, transient decreases in seminiferous height, testis weight, and sperm counts were documented after 50 mg/kg for 5 days, with recovery within 4–7 weeks; by contrast, 100 mg/kg resulted in epithelial collapse, germ cell apoptosis, abnormal sperm morphology, and LDH elevation, with only partial recovery by week 10, as reported by Prasad et al., 2010a, Prasad et al., 2010b, Prasad et al., 2011 (12, 17, 18). No significant alterations in spermatogenesis, seminiferous morphology, or testis weight were recorded in C3H/HeJ mice following 150 mg/kg in drinking water for 2 months, as observed by Schultheis et al. (2012) (16).

Overall imatinib-induced testicular toxicity exhibits both dose dependency and strain specificity, with reversible alterations at lower doses but partly irreversible damage at higher exposures; Swiss albino and Kunming mice appear particularly susceptible (**Table 1**).

#### 2.5.2 Developmental stage–dependent toxicity in rat models

There were six studies across rat models as summarized in **Table 2**. This includes one using adult female rats (20), two using Wistar (21, 22), and three using Sprague-Dawley (SD) strains (14, 23, 24), doses regimens ranged from 6 to 200 mg/kg for durations of 3–30 days. Administration was performed through oral gavage (14, 21, 23, 24), intraperitoneal injection (20), or drinking water (22), and reproductive outcomes were evaluated by histology, morphometric analysis of seminiferous tubules, sperm assessment, and hormone profiling. The age of treatment was from fetal, neonatal, juvenile and pubertal period. **Table 2** shows the species age and the corresponding human age (25).

Fetal exposure was associated with lasting alterations in the offspring. In the study by Suzan et al. (2021) (20), treatment of pregnant females on gestational days 1–8 resulted in dose-dependent reductions in sperm counts and increases in oxidative stress markers in male progeny at postnatal day 60 (PND 60), while nuclear maturation of germ cells was unaffected.

Neonatal exposure produced the most severe gonadotoxic effects. Administration of 200 mg/kg from PND1 to PND10 caused Sertoli cell vacuolization, detachment of the seminiferous epithelium, abnormal sperm morphology, and spermatid arrest, with only partial recovery observed by PND140, as reported by Al-Allaf et al. (2021) (14). Significant reductions in testis weight, seminiferous cord length, and germ cell number, together with enhanced apoptosis and persistent elevations of LH and FSH, were further demonstrated following exposure at PND5, as demonstrated by Nurmio et al. (2007) (23). In a subsequent study, decreased weights of testes, epididymides, and seminal vesicles, sustained gonadotropin elevation, and reduced litter size were still observed despite preserved adult testicular morphology and sperm counts, as confirmed by Nurmio et al. (2008) (24).

Juvenile exposure appeared less detrimental. In the study of Tauer et al. (2014) (22) induced only mild, non-significant reductions in testosterone, while inhibin B levels remained unchanged, indicating no overt impairment of spermatogenesis.

Pubertal exposure produced moderate impairments. Reductions in sperm counts, testosterone concentrations, and seminiferous tubule diameter, accompanied by elevated gonadotropin levels and thickening of the tunica albuginea, although less severe than those observed after neonatal treatment, were observed by Yaghmaei et al. (2009) (21) following 30 days of treatment with 6–12 mg/kg.

Findings from rat models demonstrated a developmental stage dependent pattern, with neonatal exposure producing the most severe injury, whereas juvenile and pubertal exposures caused milder but measurable impairments (**Table 2**).

#### 2.5.3 Evidence from Zebrafish models

Zebrafish have been widely utilized as an aquatic model to investigate imatinib-induced reproductive toxicity and potential transgenerational epigenetic effects (26). In the study by Ahmadi et al. (2024), exposure was associated with a dose-dependent reduction in gamete quality, fecundity, and subsequent offspring development. Histological analyses revealed impaired follicular maturation in females and decreased sperm motility in males, while reproductive outcomes including fertilization and hatching rates were adversely affected.

These findings suggest that zebrafish provide a sensitive system to capture both functional and molecular consequences of imatinib exposure, as summarized in **Table 4**.

#### 2.5.4 Evidence from human studies

There have been four studies on males with CML as summarized in **Table 5**. In a group aged 15–84 years, who received oral imatinib therapy at 400 mg/day for treatment durations ranging from 3 to 96 months. Reproductive outcomes were assessed through semen analysis, hormonal profiling, and imaging modalities.

Hormonal alterations were consistently recorded. Gynecomastia in the early phase of treatment, occurring in 18% of patients, together with reductions in total testosterone (>90%) and free testosterone (73%), was reported by Gambacorti-Passerini et al. (2003) (27). Elevations of progesterone and 17-hydroxyprogesterone in a subset of patients were also identified in the same study. Abnormal gonadotropin patterns, characterized by significant increases in LH and FSH with persistent reductions in testosterone, were demonstrated by Ahmed et al. (2021), who also documented a mean platelet count reduction of 14.2%, while white blood cell and hemoglobin levels remained unchanged (28). Impairments in semen quality were described by Chang et al. (2017), with a mean 22% reduction in sperm density together with declines in total sperm count, motility, and viability (13). Ultrasound examinations in the same cohort revealed enlarged hypoechoic regions in the seminal vesicles, and hydroceles were detected in 36.5% of patients. Emerging 2024 clinical evidence provides a contrasting signal to preclinical gonadotoxicity: in a matched pre/post semen analysis of men on long-term TKIs, no deterioration in key semen parameters was observed, aligning with several fatherhood reports under ongoing imatinib (29).

These findings argue that partial or full functional recovery of spermatogenesis is feasible in some adults despite prolonged exposure, though small cohorts and selection biases limit certainty and mandate long-term surveillance (**Table 5**).

#### 2.5.5 Insights from *in vitro* cell-based models

A range of *in vitro* models has been employed to delineate the cellular basis of imatinib-induced reproductive toxicity as depicted in **Table 6**. This includes mouse-derived Leydig cell lines (TM3) (30) and Sertoli cell lines (TM4) (31), Mouse spermatogonial stem cells (mGSCs) and their derivatives, as well as seminiferous tubule fragments from adult Sprague-Dawley rats and C57BL/6J mice, as summarized in **Table 6**. Drug exposure was generally applied at concentrations between 1 and 100 µM for durations from 24 hours to 10 days, and outcomes were assessed by measures of cell viability, proliferation, apoptosis, oxidative stress, growth factor secretion, and differentiation potential.

A study by Kheradmand et al. (2016) demonstrated that exposure of TM3 Leydig cells to 2.5-20 µM for up to 6 days led to a concentration-dependent decline in cell viability, with PDGF levels significantly increased and SCF levels unchanged (30). Enhanced ROS generation, lipid peroxidation, LDH release, and apoptosis, accompanied by reduced mitochondrial membrane potential and antioxidant activity, were observed in TM4 Sertoli cells, as reported by Hashemnia et al. (2016). In spermatogonial cultures, diminished proliferation and survival of differentiated c-KIT^+^ spermatogonia, together with preserved self-renewal of SSCs, were observed in a study by Heim et al. (2011), who also recorded reduced DNA synthesis and increased apoptosis (10). Organotypic cultures of seminiferous tubule segments confirmed suppressed DNA synthesis, reduced colony formation, and enhanced caspase-3 activation; partial rescue of germ-cell survival with exogenous SCF supplementation was documented by Eggert et al. (2024) (32).

*In vitro* systems revealed consistent impairment of germ-cell viability and proliferation through SCF/c-KIT inhibition, with additional oxidative and functional changes in Leydig and Sertoli cells (**Table 6**).

#### 2.5.6 Integrated mechanistic insights from multi-scale models

Imatinib exposure across *in vitro*, *in vivo*, and organotypic systems has consistently been associated with stage-specific and dose-dependent impairment of spermatogenesis. The summarized evidence in **Table 7** follows the sequential process of spermatogenesis, ranging from SSC self-renewal to meiotic progression, spermatid maturation, and interstitial support. A study by Eggert et al. (2024) demonstrated that, at the spermatogonial stem cell (SSC) level, exposure to 1–10 μM reduced DNA synthesis, S-phase fraction, and colony formation, whereas treatment at 100 μM resulted in irreversible SSC depletion, near-complete colony loss, and sustained suppression of c-KIT expression.

At the spermatocyte stage, partial disruption of the BTB, apoptosis, and reductions in sperm counts and motility at 60 mg/kg were observed by Chang et al. (2021), while complete BTB breakdown, pronounced apoptosis, and azoospermia at 120 mg/kg were also documented in the same study (19). During spermatid development and sperm maturation, progressive morphological abnormalities and motility decline under increasing doses, with partial recovery at ≤60 mg/kg but persistent impairment at ≥120 mg/kg, were reported by Chang et al. (2021). At the interstitial level, no significant changes in Leydig cell morphology or PDGF/SCF expression following 150 mg/kg were described in the study of Nurmio et al. (2007) (23), whereas interstitial edema, fibrosis, increased Leydig cell counts, and reductions in StAR and 3β-HSD expression after 200 mg/kg were reported by Al-Allaf et al. (2021) (14).

Across *in vitro*, *in vivo*, and organotypic models, a continuum of dose-dependent injury was delineated, ranging from SSC depletion to germ-cell loss, BTB disruption, and interstitial dysfunction (**Table 7**).

## 3 Discussion

### 3.1 Overall synthesis of evidence

Across 20 studies encompassing animal models, human cohorts, and *in vitro* systems, three consistent patterns emerge: dose dependency, developmental-stage sensitivity, and conditional reversibility. Imatinib-induced gonadotoxicity appears to follow a progressive continuum, beginning with SSC impairment, followed by disruption of the BTB, and culminating in defective spermatid maturation and interstitial dysfunction.

However, a key observation of this review is the apparent discrepancy between experimental and clinical evidence. While animal and *in vitro* studies consistently demonstrate pronounced gonadotoxic effects, including apoptosis, structural damage, and SSC depletion, clinical findings are more heterogeneous and, in several cases, less severe.

This divergence can be explained by multiple factors. First, experimental studies frequently employ higher or tightly controlled dosing regimens, which may exaggerate toxic effects compared with real-world clinical exposure. Second, developmental stage at exposure differs substantially, with many animal studies focusing on neonatal or prepubertal periods, whereas most clinical data derive from adult patients. Third, clinical studies are constrained by smaller sample sizes, heterogeneous treatment durations, and potential selection bias, which may obscure subtle or long-term reproductive effects.

Importantly, available human data suggest that functional fertility may be preserved in a subset of patients. Reports of stable semen parameters in some cohorts, together with documented cases of successful fatherhood during ongoing imatinib therapy, indicate that spermatogenesis may remain functionally intact despite biochemical or subclinical alterations.

Taken together, these findings indicate that imatinib-induced reproductive toxicity is highly context-dependent, shaped by the interplay of dose, developmental timing, and individual susceptibility, and should therefore be interpreted with caution when translating experimental evidence into clinical practice.

### 3.2 Mechanistic basis of imatinib-induced gonadotoxicity

Imatinib-induced testicular toxicity is primarily mediated through inhibition of the c-KIT and PDGFR signaling pathways. Suppression of these receptors has been shown to impair PI3K/AKT and MAPK signaling cascades, reduce BCL-2 expression, and activate caspase-3, thereby promoting spermatogonial apoptosis (23, 24, 32, 33). In addition, inhibition of PDGFR has been associated with impaired proliferation of peritubular myoid cells and reduced differentiation of Leydig cells, resulting in disruption of the seminiferous microenvironment and diminished endocrine support required for spermatogenesis (23).

Although peritubular myoid (PTM) cells are not directly involved in germ cell differentiation, they are essential for maintaining the structural integrity and functional microenvironment of the seminiferous tubules. Their roles in extracellular matrix organization, tubular contractility, and paracrine signaling contribute to the regulation of Sertoli cell function and germ cell development. Notably, PDGFR is highly expressed in PTM cells, rendering them susceptible to imatinib-mediated inhibition. Consequently, impairment of PTM cell function may indirectly disrupt spermatogenesis through alterations of the seminiferous niche and intercellular signaling environment. These alterations, together with direct effects on germ cells, contribute to meiotic arrest, defective spermatid maturation, and progressive sperm loss.

The blood–testis barrier (BTB) represents a critical structural and functional component of the seminiferous epithelium, in which an immune-privileged environment is established to support spermatogenesis. By separating the basal and adluminal compartments, the BTB protects developing germ cells from systemic immune surveillance and toxic insults. Disruption of BTB integrity has been implicated in a range of pathological conditions, including infection, inflammation, toxicant exposure, and idiopathic male infertility, and is commonly associated with germ cell loss, meiotic arrest, and impaired sperm quality. Under imatinib exposure, downregulation of tight junction proteins such as occludin, claudin-11, and Mfsd2a has been observed, accompanied by widening of intercellular junctions and discontinuity of the basement membrane (19, 20). These structural alterations facilitate intratesticular drug accumulation, promote germ cell apoptosis, and impair spermatogenesis, with corresponding reductions in semen quality reported in clinical settings (13, 23, 32). Thus, BTB disruption should be regarded not only as a structural abnormality but also as a functionally significant event contributing directly to male reproductive impairment.

Endocrine and microenvironmental disturbances further reinforce these processes. Reductions in testosterone levels, together with compensatory elevations of luteinizing hormone (LH) and follicle-stimulating hormone (FSH), have been consistently reported in both experimental models and clinical cohorts (27, 28).Impaired steroidogenesis in Leydig cells reduces intratesticular androgen levels, while Sertoli cell dysfunction compromises germ cell survival (23, 30). *In vivo* studies have demonstrated long-term depletion of spermatogonial stem cells, shortening of seminiferous cords, and reduced litter size following early-life exposure (23, 24). Clinical observations of delayed puberty and reduced testicular volume in adolescents further support increased vulnerability of spermatogonial stem cells within an immature microenvironment.

**Figure 2:**
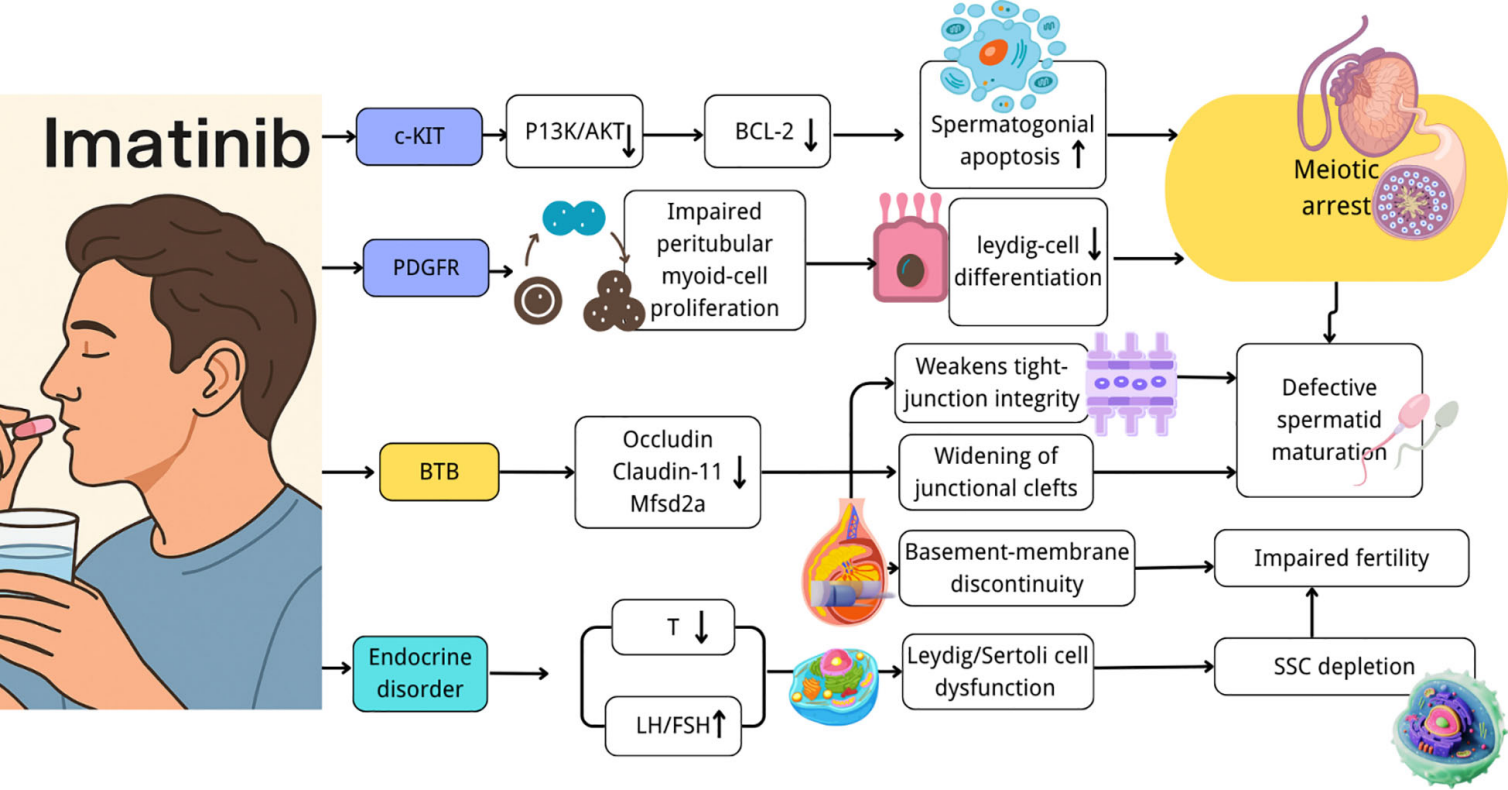
Mechanistic framework of imatinib-induced testicular toxicity and male fertility impairment. Hormones: FSH, Follicle-Stimulating Hormone; LH, Luteinizing Hormone; T, Testosterone. Structural & Administration Terms: BTB, Blood -Testis Barrier; SSC, Spermatogonial Stem Cells; i.p., Intraperitoneal. Signaling & Molecular Markers: c-KIT, Stem Cell Factor Receptor; PDGFR, Platelet-Derived Growth Factor Receptor; PI3K/AKT, Phosphatidylinositol 3-Kinase/Protein Kinase B Pathway; BCL-2, B-Cell Lymphoma 2. Tight Junction Proteins: Occludin; Claudin-11, Mfsd2a (Major Facilitator Superfamily Domain-Containing Protein 2a.

Taken together, these mechanisms form an integrated pathogenic cascade in which spermatogonial stem cell impairment, BTB disruption, and endocrine imbalance collectively result in defective spermatogenesis and male infertility (**Figure 2**).

### 3.3 Developmental stage and dose dependency

Both developmental timing and cumulative dose critically determine the extent and reversibility of gonadal injury. Rodent studies indicate that neonatal and pubertal exposures-periods of SSC expansion, BTB establishment, and HPG-axis maturation-produce lasting spermatogenic defects, whereas adult exposure is generally followed by recovery after withdrawal (10, 14, 28). Clinical data align, with adolescents on long-term therapy showing delayed puberty, reduced testicular volume, and incomplete virilization (13, 22, 28).

A graded dose-response has also been observed: low doses allow spermatogenic recovery, moderate doses yield partial restoration, and high or prolonged exposures result in irreversible SSC depletion and fibrosis (10, 14, 17, 20, 23, 28, 30). Clinical cohorts corroborate associations between cumulative dose, sperm density, and pubertal delay (13, 27, 28), though strain- and route-specific differences highlight interspecies variability (16).

Collectively, toxicity can be conceptualized within a two-dimensional framework defined by developmental stage and cumulative exposure. High-dose or early-life exposures constitute the highest-risk quadrant, often leading to irreversible infertility, whereas adult low-dose exposures typically permit functional recovery. This model provides a basis for risk stratification and fertility-preservation planning, although standardized thresholds remain to be defined.

### 3.4 Methodological heterogeneity and translational implications

Methodological heterogeneity across studies represents a major source of variability and is central to explaining the discrepancies between experimental and clinical findings. Differences in administration routes, dosing regimens, exposure duration, and developmental timing substantially influence both the severity and reversibility of reproductive toxicity. In preclinical models, imatinib is typically administered under tightly controlled conditions—often via intraperitoneal injection or oral gavage—ensuring consistent systemic exposure but not fully reflecting the pharmacokinetic variability seen in clinical practice. Moreover, experimental regimens frequently involve higher or more acute dosing relative to clinically equivalent exposure, particularly in short-term high-dose designs, thereby amplifying observed toxic effects.

By contrast, human exposure generally involves standardized oral dosing (e.g., 400 mg/day), prolonged treatment, and interindividual variability in absorption and metabolism (27). These conditions may permit partial physiological adaptation, potentially attenuating overt gonadotoxic effects. Developmental stage further accentuates these differences. Animal studies often target neonatal or prepubertal periods, when SSCs and BTB formation are highly vulnerable, whereas most clinical cohorts consist of adult patients with established spermatogenesis. This discrepancy likely contributes to the relatively milder or reversible reproductive effects observed in humans compared with preclinical models (14).

Additionally, heterogeneity in outcome assessment—including histological endpoints in animal models versus semen analysis and hormonal profiling in clinical studies—limits direct comparability. The small sample sizes and predominantly retrospective design of clinical studies further reduce statistical power and increase susceptibility to bias. Collectively, these methodological differences suggest that preclinical findings may overestimate the magnitude of gonadotoxicity when directly extrapolated to clinical settings.The limited availability of high-quality clinical data further constrains the strength of translational conclusions.Future studies should therefore prioritize alignment of experimental designs with clinically relevant dosing strategies and exposure conditions, and incorporate standardized reproductive endpoints to enhance translational validity and support evidence-based fertility counseling.

### 3.5 Clinical evidence and implications for fertility counseling

Clinical evidence on male reproductive outcomes during imatinib therapy remains limited but reveals a heterogeneous pattern that contrasts with the more consistent gonadotoxic effects observed in experimental models. While several studies report reductions in testosterone levels, altered gonadotropin profiles, and impaired semen parameters, these findings are not universally observed. Importantly, available data suggest that fertility potential is preserved in a subset of adult patients. Reports of successful fatherhood during ongoing imatinib therapy, together with longitudinal observations demonstrating stable semen parameters in some cohorts, indicate that spermatogenesis may remain functionally intact despite biochemical or subclinical alterations.

In contrast, younger patients appear to be more vulnerable, particularly during prepubertal and pubertal stages. Clinical observations of delayed puberty, reduced testicular volume, and impaired steroidogenesis suggest that disruption during these critical developmental windows may have long-term consequences. These findings have direct implications for clinical practice. For adult patients, current evidence does not support a uniform recommendation to discontinue imatinib solely based on concerns of infertility. Instead, individualized evaluation—including semen analysis and hormonal assessment, should guide fertility counseling. For adolescents and children, a more precautionary approach is warranted. Early counseling regarding potential reproductive risks and timely consideration of fertility preservation strategies are essential. Sperm cryopreservation remains the standard option for postpubertal males, whereas experimental approaches such as spermatogonial stem cell or testicular tissue preservation may be considered for prepubertal patients. Overall, the available evidence suggests that imatinib-associated reproductive toxicity is variable, potentially reversible in adults, but more uncertain and potentially severe in younger populations, underscoring the importance of individualized risk assessment and long-term follow-up.

### 3.6 Reversibility of gonadal and endocrine function

Partial but conditional recovery of gonadal function has been observed following discontinuation of imatinib. The extent of recovery is thought to depend on the preservation of spermatogonial stem cells (SSCs) and the functional resilience of the hypothalamic–pituitary–gonadal (HPG) axis, with outcomes influenced by developmental stage, cumulative dose, and duration of exposure.

The integrity of SSCs is considered a key determinant of spermatogenic recovery after cytotoxic injury (34, 35). In murine models, recovery efficiency has been shown to depend on the kinetics of SSC repopulation following treatment-induced depletion (15, 35) When exposure occurs during the prepubertal period, disruption of SSC quiescence and accelerated depletion are more likely to result in irreversible spermatogenic failure. In contrast, postpubertal or adult exposure has been associated with partial recovery, likely mediated by activation of residual SSCs and remodeling of the local microenvironment (24, 36–38). Clinical observations are generally consistent with these findings, with improvements in semen parameters reported in a subset of patients after treatment withdrawal, suggesting the persistence of functional SSCs under supportive conditions (15, 39).

Endocrine recovery has also been described, although with considerable variability. Imatinib-induced alterations of the HPG axis are typically characterized by reduced testosterone levels accompanied by compensatory elevations in luteinizing hormone (LH) and follicle-stimulating hormone (FSH) (27, 28). In rodent models, gonadotropin levels have been observed to gradually normalize following drug withdrawal, although recovery may be prolonged, particularly after early-life exposure (22, 24). In human cohorts, recovery has been inconsistent. Improvements in hormonal profiles and semen quality have been reported in some patients after prolonged discontinuation (13, 15, 40), whereas persistent oligozoospermia or endocrine dysfunction has been observed in others despite cessation (41). These findings suggest that recovery of the HPG axis is strongly influenced by developmental timing and cumulative exposure (15).

Overall, recovery of reproductive function following imatinib exposure appears to be incomplete and variable across individuals. The extent of SSC preservation, timing of exposure, and cumulative drug burden are likely to play critical roles. These considerations underscore the importance of long-term endocrine and reproductive monitoring, as well as early fertility counseling in clinical practice.

### 3.7 Individualized predictive biomarkers for clinical risk stratification

Although diverse mechanisms of imatinib-induced gonadotoxicity have been described, clinical translation remains limited by the absence of individualized predictive biomarkers. The identification of such markers is considered essential for early recognition of high-risk patients and for the timely implementation of fertility-preserving strategies. Available evidence supports three principal categories of predictive indicators, including endocrine markers, circulating molecular signatures, and pharmacokinetic or pharmacogenomic determinants, reflecting a continuum from clinically validated measures to experimental approaches with translational potential.

Endocrine alterations remain the most established indicators of gonadal impairment during tyrosine kinase inhibitor therapy. Reductions in testosterone accompanied by elevations in luteinizing hormone (LH) and follicle-stimulating hormone (FSH) have been observed in both clinical cohorts and experimental models, reflecting compensatory disruption of the hypothalamic–pituitary–gonadal (HPG) axis (42). Inhibin B, either alone or in combination with the FSH/inhibin B ratio, has been recognized as a surrogate marker of Sertoli cell function and spermatogenic reserve, with reductions reported in adolescents receiving TKI therapy (43, 44). Additional evidence from pediatric cancer survivors indicates that Leydig cell dysfunction is frequently present, characterized by a mild decline in serum testosterone accompanied by a disproportionate increase in LH, which has been interpreted as a compensatory response to impaired Leydig cell activity (27, 45). These endocrine disturbances have been associated with hypogonadism and delayed pubertal onset (46, 47). while reduced testicular volume and delayed sexual maturation have been observed in a subset of patients, suggesting concomitant impairment of spermatogenesis (48). Collectively, combined assessment of LH, FSH, testosterone, and inhibin B is considered a feasible approach for individualized risk stratification, particularly in pediatric and adolescent populations at increased risk of long-term reproductive sequelae.

Circulating microRNAs (miRNAs) provide an additional layer of molecular information and have been proposed as non-invasive biomarkers of early testicular injury. Their stability in plasma and serum is maintained through encapsulation within extracellular vesicles or binding to RNA-associated proteins, enabling reliable detection in peripheral blood (49–53). Several miRNAs, including miR-34c, miR-15a, and miR-21, are known to regulate spermatogenesis and germ cell apoptosis, and altered expression patterns have been documented in infertile men and in experimental models of testicular dysfunction (54–56). Distinct miRNA signatures have also been reported to differentiate obstructive from non-obstructive azoospermia and to reflect the severity of spermatogenic failure (57). However, systematic evaluation of these biomarkers in patients receiving imatinib remains lacking. Future studies should therefore focus on integrating miRNA profiles with endocrine and oxidative stress markers to improve predictive performance.

Pharmacokinetic and pharmacogenomic determinants represent an additional source of interindividual variability in susceptibility. Therapeutic drug monitoring studies have demonstrated that higher imatinib trough concentrations (C_min_) are associated with both increased toxicity and improved clinical response in chronic-phase CML and gastrointestinal stromal tumors (60). The ABCG2 c.421C>A polymorphism has been linked to elevated plasma trough levels, indicating greater drug exposure in carriers of the A allele compared with those carrying the C allele (58).

Although direct causal relationships between these parameters and gonadal impairment remain to be established, their biological plausibility and clinical accessibility support their incorporation into predictive frameworks.

An integrated model combining endocrine markers (LH, FSH, testosterone, inhibin B), molecular signatures (circulating miRNAs), and pharmacokinetic or pharmacogenomic indicators (C_min_, ABCG2 genotype) may enhance individualized risk stratification. Such an approach may facilitate early fertility counseling, inform dose optimization, and support protective interventions aimed at preserving reproductive potential without compromising oncologic efficacy.

### 3.8 Genotoxicity and non-reproductive adverse effects

Current evidence indicates that paternal exposure to imatinib is associated with minimal genotoxic risk. Systematic analyses of pregnancies fathered by men receiving imatinib have not demonstrated increased rates of congenital anomalies compared with background population levels. Reviews have similarly concluded that paternal use of tyrosine kinase inhibitors, including imatinib, does not significantly impair fertility or offspring health.

Beyond reproductive outcomes, imatinib therapy has also been associated with non-gonadal toxicities. Testicular hydrocele and systemic fluid retention have been recurrently reported and are considered part of a broader spectrum of fluid retention–related adverse events. These effects are thought to arise from off-target inhibition of c-KIT and PDGFR signaling, which play key roles in vascular integrity, interstitial fluid regulation, and extracellular matrix remodeling (59, 60).

Overall, the toxicological profile of imatinib extends beyond gonadal endpoints, highlighting the need to incorporate systemic adverse effects into long-term safety assessments.

### 3.9 Clinical strategies and fertility preservation

Recent expert recommendations have emphasized the integration of fertility counseling into standard care pathways for patients with chronic myeloid leukemia (CML) (61). The 2025 European LeukemiaNet (ELN) guidelines, published in *Leukemia*, highlight emerging evidence on TKI-associated reproductive risks and recommend individualized counseling prior to conception attempts or treatment interruption. This shift reflects the growing recognition that fertility preservation should be considered an integral component of comprehensive CML management, particularly in patients of reproductive age.

Given the partially reversible nature of imatinib-induced gonadotoxicity, clinical management is aimed at preserving reproductive potential while maintaining oncologic efficacy. Strategies to reduce cumulative exposure include treatment-free remission (TFR) in patients achieving deep molecular responses, as well as experimental intermittent dosing regimens designed to allow partial recovery of spermatogonial stem cells (SSCs). Improvements in semen quality following TKI discontinuation have been described in case reports; however, robust evidence from prospective TFR cohorts remains limited (41, 62–64). Intermittent dosing approaches have also been proposed, although these remain experimental and lack prospective validation (65, 66).

In pediatric CML, dose individualization is particularly important because of age-dependent pharmacokinetics and the need to minimize long-term toxicity. Standard regimens of 260–340 mg/m² per day (equivalent to 400–600 mg in adults) have been widely used, and intermittent schedules have been explored in some cohorts to mitigate effects on growth and development (67). Nevertheless, endocrine abnormalities and gonadal toxicity continue to be observed in long-term follow-up, underscoring the importance of systematic endocrine monitoring (64, 68). For patients with intolerance or significant toxicity, second-generation TKIs such as dasatinib may achieve faster and deeper responses; however, their long-term reproductive safety has not been fully established (69).

Protection during puberty and adolescence remains critical, as prepubertal exposure is associated with the highest risk of irreversible SSC depletion and delayed gonadal development (23). When clinically appropriate, delaying treatment until after pubertal onset or implementing short-term discontinuation under close molecular monitoring may provide opportunities for fertility preservation (64). Fertility counseling should therefore be systematically incorporated into clinical care, including semen cryopreservation for pubertal and adult males and experimental testicular tissue cryopreservation for prepubertal boys (70). Many of these strategies remain investigational, highlighting the need for further clinical validation.

### 3.10 Research gaps and future directions

Despite the availability of several potential protective strategies, substantial gaps remain in the understanding and management of imatinib-induced gonadotoxicity. These gaps can be broadly categorized into clinical, developmental, mechanistic, and infrastructural domains.

Long-term, adequately powered cohort studies in pediatric and adolescent populations are lacking, resulting in limited characterization of fertility trajectories following early-life exposure to TKIs (64). The reproductive effects of treatment-free remission (TFR) and second-generation TKIs remain insufficiently defined. Prospective evaluation of fertility outcomes during TFR, together with systematic safety assessment of newer agents, is therefore required (53, 70).

Prepubertal and pubertal periods remain insufficiently studied. Exposure during these stages has consistently been associated with a higher risk of irreversible SSC depletion and delayed gonadal maturation (23). Strategies such as delaying therapy until after pubertal onset or implementing short-term discontinuation have been proposed but remain experimental and require rigorous validation. Fertility preservation should be systematically incorporated into care, including semen cryopreservation and experimental testicular tissue preservation (41).

Approaches aimed at preserving fertility, including stem cell factor analogues, antioxidants, and niche-supporting compounds, have shown promise in preclinical studies. However, none have yet been validated in clinical settings, highlighting the need for translational research.

Emerging techniques such as single-cell transcriptomics and spatial profiling provide high-resolution insights into SSC niche dynamics, BTB integrity, and intercellular signaling pathways disrupted by imatinib (36, 37). These technologies are expected to facilitate translation of mechanistic findings into clinical applications.

The absence of dedicated clinical registries limits systematic evaluation of reproductive outcomes, including semen quality, endocrine recovery, pubertal development, and potential epigenetic effects in offspring. Establishment of such registries will be essential for the development of evidence-based guidelines.

## 4 Conclusion

Available evidence indicates that imatinib exerts gonadotoxic effects in a dose- and age-dependent manner. The principal mechanisms involve inhibition of c-KIT and PDGFR signaling, disruption of the blood–testis barrier, and alterations in the hypothalamic–pituitary–gonadal axis. Both experimental and clinical data identify neonatal and prepubertal periods as particularly vulnerable developmental windows, during which exposure is frequently associated with partially irreversible impairment of spermatogenesis and endocrine function.

Although partial recovery of gonadal and hormonal function has been observed in some settings, long-term reproductive outcomes remain insufficiently characterized, particularly in pediatric and adolescent populations. With improving survival in CML, increasing attention is being directed toward fertility preservation and individualized treatment strategies.

## Data Availability

The original contributions presented in the study are included in the article/**Supplementary Material**. Further inquiries can be directed to the corresponding author.
