# Supplementary material for: A scoping review of imatinib-induced testicular toxicity and male fertility impairment
Source: Front Endocrinol (Lausanne). 2026 May 14;17:1811653. doi: 10.3389/fendo.2026.1811653 (PMC13218081; doi:10.3389/fendo.2026.1811653)
Supplement: Supplementary file 1 [file Table1.docx]

**PUBMED：**

("Imatinib"[Mesh] OR "Imatinib mesylate"[Title/Abstract] OR "Imatinib"[Title/Abstract])

AND

("Testis"[Mesh] OR "Testes"[Title/Abstract] OR "Spermatogenesis"[Mesh] OR "Spermatogenesis"[Title/Abstract]

OR "Sperm"[Title/Abstract] OR "Male fertility"[Title/Abstract]

OR "Leydig cells"[Title/Abstract] OR "Sertoli cells"[Title/Abstract]

OR "Male reproductive toxicity"[Title/Abstract])

**Scopus**

TITLE-ABS-KEY (imatinib OR "imatinib mesylate")

AND

TITLE-ABS-KEY (testis OR testes OR spermatogenesis OR sperm OR "male fertility"

OR "leydig cells" OR "sertoli cells" OR "male reproductive toxicity")

**Web of Science**

TS=(imatinib OR "imatinib mesylate")

AND

TS=(testis OR testes OR spermatogenesis OR sperm OR "male fertility"

OR "leydig cells" OR "sertoli cells" OR "male reproductive toxicity")
